# A Confederate Veteran’s Thoughts on the Blue and Grey

**Published:** 1898-07

**Authors:** 


For the Texas Medical Journal.

### A Confederate Veteran's Thoughts on the Blue and Grey.

[Read at a reunion of C. S. A. Veterans at Calvert, Texas, May 31, 1898, by Dr. Daniel Parker, and dedicated to his old comrades wherever they may be.]

Now don't be crying, mother,
You should be proud of Ben,
He looked, of all, the proudest
Among a thousand men.
It set me straight to thinking
How I too marched away,
To join the noble army
Dressed in Confed'rate grey.

I somehow felt like fighting
When first our Ben marched out—
Right dress, front face, a standin'
So tall and straight and stout.
But I cannot like the color
Of the clothes he wore that day.
'Twould suit me whole lots better
If they looked a little grey.

But he don't know the dif'rence
I 'spose he likes the blue,
Though as for me I can't forget
I fought it four years through.
And though I am plum willin'
For Ben to have his way,
I just could fight lots better
If my clothes were sort o' grey.

The flag's all right, I like it,
It must not ever fall,
I sure would like to plant it
On Moro Castle's wall,
And I know that I could do it,
In the good old fashioned way,
If I had a few old comrades,
Dressed in Confed'rate grey.

They say its hot in Cuba,
And when the boys get there
They'll shuck those hot blue fixins,
And canvas suits will wear.
I like that plan amazin',
For when they march a day,
The dust and dirt will change 'em
To the good old fighting grey.

This is a great big country,
And when it comes to blows,
We'll keep the world from treading
On Uncle Samuel's toes.
But if I should take a rifle,
I've just got this to say:
I could handle her whole lots better,
If my clothes were sort o' grey.

It breaks my heart, old woman,
To hear you sobbing so,
I lived through four years fighting,
Ben's coming back I know,
But when he goes to lying,
(As he's almost sure to do)
About the gallant fighting,
Of our volunteers in blue,
Why then, why then—confound him,
I'll have a word to say,
And beat him with one bigger,
About the boys in grey.

